# Effects of nest-box environment on fledgling success rate and pathogen load

**DOI:** 10.1017/S0031182022000695

**Published:** 2022-08

**Authors:** James F. Scott-Baumann, Eric R. Morgan, Tristan A. Cogan

**Affiliations:** 1 Institute of Biological, Environmental and Rural Science, Aberystwyth University, Aberystwyth, Ceredigion SY23 3DA, UK; 2 School of Biological Sciences, Queen's University Belfast, Belfast, UK; 3 Bristol Veterinary School, University of Bristol, Bristol, UK

**Keywords:** Bacteria, blue tit *Cyanistes caeruleus*, fledging success, fungi, great tit *Parus major*, nest box, parasite

## Abstract

Nest boxes have been used for many decades as tools for conservation and to study avian population dynamics. Plastic is increasingly used as a material for nest boxes, but no studies have investigated effects of this different material. Two consecutive studies were conducted to investigate effects of nest-box environment on nidicolous parasites, bacteria and fungi, as well as nest success, in blue tits *Cyanistes caeruleus* and great tits *Parus major*. The first compared microclimate and parasite and pathogen load in plastic and wooden nest boxes. The second tested the nest protection hypothesis – that birds naturally incorporate aromatic herbs into nests to decrease nest parasites and pathogens – by comparing parasite and pathogen load in plastic nest boxes to which aromatic or non-aromatic plant material was added. No significant difference in nest-box temperature or relative humidity was found between plastic and wooden boxes. Wooden boxes, however, contained 30-fold higher numbers of fleas and a higher total bacterial load on chicks. Fledging success for blue tit broods was significantly higher in wooden boxes. Parasites and bacteria did not decrease by the inclusion of aromatic herbs. The results increase the evidence base for nest-box design in support of plastic, which can provide an appropriate alternative nest-box material to wood, with apparently no difference in microclimate and no increase in the load of measured parasites and pathogens.

## Graphical abstract

**Figure:**
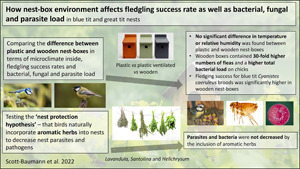

## Introduction

Wooden nest boxes have been used in conservation for many years, for example since 1947 in the well-known Wytham Woods study site in Oxford (Perrins and Overall, 2001). In the last decade or so, new nest-box styles have become commercially available (Pearce Environment, 2014). Plastic, being cheaper, recyclable and longer lasting, could be a more sustainable nest-box material compared to wood. Despite these new nest-box styles having been widely available for several years, few published studies have investigated either the microclimate inside these boxes or how this might affect nest site choice, or indeed any variation in breeding success or nidicolous parasite and pathogen levels associated with nest-box material. Those studies that have been carried out have focussed on wood-crete boxes; another new nest-box material which is made from a blend of cement and sawdust (Browne, 2006; Garcia-Navas *et al*., 2008). Many species of parasite are commonly found to affect birds nesting in artificial nest boxes, including fleas (Richner *et al*., 1993), mites (Gwinner *et al*., 2000), blowfly larvae (Hurtrez-Bousses *et al*., 1997) and ticks (Goodenough and Hart, 2012), and are all known to be pathogenic to chicks. Wild birds are also a known reservoir for multiple antibiotic resistant forms of bacteria, including Enterobacteriaceae expressing extended spectrum beta-lactamases, which could pose a threat to humans and the commercial poultry farming industry through the transmission of drug-resistant bacteria (Tsubokura *et al*., 1995; Silva *et al*., 2011; Hasan *et al*., 2012; Poirel *et al*., 2012; Tausova *et al*., 2012). The way in which nest boxes are used and managed is therefore potentially important not only for the target avian populations, but also for ecosystem health and the security of human health and food production systems.

A previous study comparing occupancy rates in nest boxes of different material showed that birds typically preferred nesting in wood-crete boxes over wood (Browne, 2006). A different study showed that wood-crete boxes typically had earlier clutches and shorter incubation times, leading to more reproductive efforts per season. This was attributed to the temperature difference, which was on average 1.5°C higher in wood-crete boxes (Garcia-Navas *et al*., 2008). However, this temperature increase could equally be detrimental to egg development, depending on external environmental temperatures. Garcia-Navas *et al*. (2010) later found that chicks in wood-crete nest boxes were lighter, which could be due to greater transpiration through panting and other mechanisms used for cooling by chicks. Other studies have shown that differences in temperature, as a result of nest-box orientation, also affect occupancy rates (Ardia *et al*., 2006; Goodenough *et al*., 2008). Nest-box microclimate is clearly an important factor in brood success, which needs to be investigated for plastic boxes. The British Trust for Ornithology (BTO) currently recommends against the use of plastic boxes on its website (BTO, 2013), due to over-heating and condensation issues. There is, however, no published scientific data comparing plastic nest boxes with other types, in terms of temperature or humidity, or effects on chick health and nest success.

Several species of birds, including blue tits *Cyanistes caeruleus* have been shown to regularly incorporate fresh green plants into their nests, after nest building has ended (Wimberger, 1984; Clark and Mason, 1985; Lambrechts and Dos Santos, 2000; Mennerat *et al*., 2009*b*). Various hypotheses exist regarding this behaviour, the most accepted of which is the nest protection hypothesis (NPH), which states that phytochemical compounds from the plants decrease nest pathogens, indirectly benefitting chicks (Clark and Mason, 1985). Typically seen in cavity nesting species rather than open-cup nesters (Clark and Mason, 1985), the behaviour has also been shown to be more likely to occur in birds that re-use their nests, rather than those that build new ones (Wimberger, 1984; Clark and Mason, 1985). These birds would be expected to face higher parasite and bacterial burdens through overwintering of parasites and contamination of the previous season's nests, and therefore make the evolution of this behaviour more likely. The behaviour has mainly been observed in birds nesting in artificial nest boxes. The plants are actively sought out by birds, being included in much higher proportions than if all plants in the nest vicinity are randomly sampled (Clark and Mason, 1985). They are also typically aromatic in nature (Lambrechts and Dos Santos, 2000; Petit *et al*., 2002) and many have been shown to have antibacterial (Sivropoulou *et al*., 1995; Rossi *et al*., 2007) or antiparasitic effects (Perrucci *et al*., 1995; George *et al*., 2008). All these factors lend circumstantial support to the NPH, i.e. that these aromatic herbs provide some beneficial effects to chicks indirectly, by alleviating nest pathogens (e.g. parasites or bacteria) or ameliorating their effects.

The present study aimed to investigate the differences in nest microclimate between plastic and wooden nest boxes as well as any variation in parasite, bacterial and fungal load and nest-box success. The effect of adding ventilation holes to the microclimate of plastic nest boxes was also investigated. It also set out to challenge the NPH in blue tits, and whether this behaviour could attenuate increased infection pressure with nidicolous parasites and bacteria in nest boxes.

## Materials and methods

### Study site

The study site was Betty Daw's Wood, Gloucestershire, UK (51°57N, 002°26W), one of the longest-running nest-box sites in the UK and has contained wooden nest boxes for 50 years, which are nested in by a mixture of blue tits and great tits *Parus major* (Cumber, 1964). In 2011, 46 plastic nest boxes were erected alongside the 20 pre-existing wooden boxes. All plastic boxes had the same dimensions (width = 14 cm, depth = 16 cm, height at front = 21 cm, height at back = 24 cm, hole diameter = 0.3 cm), while wooden box dimensions varied slightly (mean width = 15 cm, s.d. = 0.7, mean depth = 17 cm, s.d. = 0.9, mean height at front = 21 cm, s.d. = 1.8, mean height at back = 24 cm, s.d. = 2.5, mean hole diameter = 0.3 cm, s.d. = 2.2). Placement heights also varied (mean = 195 cm, s.d. = 11.72), and all boxes were oriented to face north-east (mean degrees NE = 42.3°, s.d. = 22.76°). All boxes are monitored annually to determine dates and frequencies regarding clutch sizes, brood sizes and fledging rates. Prior to the 2014 breeding season, ventilation holes were drilled into 22 randomly selected plastic boxes (5 holes, 5 mm wide, in a straight line across the top of both of the 2 side walls); this provided 20 wooden boxes, 22 plastic ventilated and 24 plastic non-ventilated boxes for comparison.

### Investigating effects of nest-box type

Prior to nest building in 2014, measures of temperature and relative humidity were taken from a random subset of the plastic and wooden nest boxes, using a humidity and temperature gauge (RS Components, Corby, UK, model 1367, resolution 0.1°C/0.1%). During the 2014 breeding season nest boxes were inspected every other day and when chicks were 10 days old, 2 in each nest box were randomly selected and swabbed, just prior to ringing, for bacterial and fungal quantification, along with the walls of their nest box. Chicks were swabbed using a standardized protocol, for 10 s, rotating the swab over 2 cm^2^ of the bird's flank, taking care to avoid contamination from the cloacal region, for later bacterial quantification. Nest boxes were also swabbed for 10 s on the interior wall, 2.5 cm above where the nest matter lay. A pre-moistened Liquid Amies Elution swab collection preservation system (ESwab, Copan, Murrieta, CA) was used. After successful fledging or nest failure, all nest material was removed and placed into a sealed plastic bag. All nest removal and manipulation were carried out by a licensed bird ringer with the appropriate Natural England licences and in accordance with UK law.

Parasites were separated from the nest using Berlese (or Tullgren's) funnels. Nests were kept in sealed funnels over a mesh, with a collecting jar underneath the funnel for 10 days at room temperature in low light. Nests were agitated and had CO_2_ exhaled onto them daily to mimic the host in order to stimulate hatching of parasites, which was necessary for the extraction process. After 10 days, heat and light was applied to the nest matter using a 40 W incandescent light bulb. Nests were heated for 24 h so nest matter dried out fully to ensure that remaining arthropods were driven out of the nest material and down the funnel into the collection jar.

To quantify fungal load from swabs, 10-fold serial dilutions were prepared in saline from 10^0^ to 10^−8^ for every swab within 24 h of collection. These were then plated onto Rose Bengal Chloramphenicol agar plates (Oxoid, Basingstoke, UK, 10 × 90 mm^2^) to encourage the growth of yeasts and moulds. The swabs were then frozen for later DNA processing. The serial dilutions allowed the counting of individual colony forming units (CFUs). The plates were divided into 1/8 segments and a single drop (20 *μ*L) from each serial dilution was placed onto each of the segments, before being incubated at ambient room temperature for 7 days in sealed bags. Individual yeast CFUs were then counted and colonies identified to a crude taxonomic level in each section. The presence of any mould CFUs was also noted, but CFUs were not counted as the prevalence on 1 plate was never greater than 1. Real-time quantitative polymerase chain reaction (qPCR) was used to determine the bacterial load of swabs. DNA was extracted from swab fluid saline using Qiaxtractor-Pure Advantage version 4.15.1 (Qiagen) as per the manufacturer's standard protocol. For PCR, each reaction mixture contained 25 *μ*L [12.5 *μ*L master mix, 1 *μ*L (100 pmol) of each forward and reverse primer, 5 *μ*L nuclease-free water, 0.5 *μ*L SyBr Green, 5 *μ*L DNA template]. For total bacteria, a set of 16S-directed primers was used: forward primer: 5′ GCAGGCCTAACACATGCAAGTC 3′; reverse primer: 5′ CTGCTGCCTCCCGTAGGAGT 3′, with the following thermal cycles: 95°C for 10 min followed by 50 cycles of 95°C for 30 s and 55°C for 1 min. For Enterobacteriaceae primer, sequences were: forward primer: 5′ ATGGCTGTCGTCAGCTCGT; reverse primer: 5′ CCTACTTCTTTTGCAACCCACTC, and the thermal cycles were 95°C for 10 min followed by 45 cycles of 95°C for 15 s and 60°C for 1 min. The master mix was Brilliant II QPCR Master Mix (Agilent Technologies, Stockport, UK, #600804). PCR was performed using a Strategene Mx3005P qPCR system with MxPro software.

Hatching success percentage (ratio of maximum clutch to brood size), fledging success percentage (ratio of hatched to fledged) and occupancy rates for each nest box used over the last 4 years were calculated using data collected from 2011 to 2014. Based on the numbers of eggs and chicks observed on different dates, accurate estimates could be made of clutch size, hatchling number and number fledged. Methods of identification were based on those of the BTO Nest Record Scheme (BTO, 2014).

### Investigation of the NPH

All plastic nest boxes were visited every 3 days from the start of the breeding season (April 2013) and randomly assigned to a herb or control group when nest building began. Once the nest cup was formed, 1 g each of *Lavandula stoechas*, *Santolina chamaecyparissus* and *Helichrysum italicum* leaves was crushed and placed under the cup to avoid removal by, and any disturbance of, the birds. These species were chosen as having all been naturally introduced by blue tits into nests in other regions (Lambrechts and Dos Santos, 2000; Petit *et al*., 2002; Mennerat *et al*., 2008; Mennerat *et al*., 2009*a*). Moss collected from the trunk of a nearby tree was added to control nests in the same way. Sterile, powder-free plastic gloves were worn during this process. Adding of herbs continued along with observations every 3 days until egg laying began, at which point no more plants were added. Bacterial and fungal loads were quantified at day 10, using the method described above. Nests were removed for analysis of parasite loads, after successful fledging or nest failure occurred. With the regularity of visits this was likely to be the day of, or the day after, actual fledging. Ectoparasites were separated from the nest material as described above, counted and identified to a crude taxonomic level. Real-time qPCR was performed to assess relative numbers of total bacteria and Enterobacteriaceae on swabs, using the method described above.

### Statistical analysis

Results were analysed using IBM SPSS Statistics 21 software (IBM, New York, USA). From the log 2-based *Ct* values produced by the real-time PCR, the following formula was used to transform *Ct* values into linear indicators of bacterial abundance in arbitrary units:
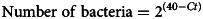


Most variables satisfied the condition that 2/3 of the points lay within 1 standard deviation either side of the mean, and were treated as if normally distributed. Those that did not, such as the bacterial and parasite data, were log-transformed prior to analysis in order to stabilize the variance. Nested analyses were performed initially on bacterial levels from chick swabs, specifying the boxes as being nested randomly within the herb groups. Due to limited sample size, nested analyses of the effects of different factors at treatment, nest box and individual bird level were not possible. Instead, non-nested designs were used, these were still valid tests with no pseudoreplication, as the dependent variables were independent of each other and did not repeat. The difference in hatching and fledging success between plastic and wooden boxes was assessed using *χ*^2^ test. Independent sample *t*-tests were performed to assess differences in means between herb and control groups, for various parameters. One-way analysis of variances compared means among plastic non-ventilated, plastic ventilated and wooden boxes; Tukey's post-hoc tests were used, and standardized residuals appeared normally distributed for all. Spearman's rank correlations were used to test correlations between all variables. All tests were 2-tailed. Factors which had univariate correlations or *a priori* reason to be tested were then included in a general linear model for each of the dependent variables of interest. Multivariate models were used to account for potentially confounding interactions between variables, such as species of bird and brood size, as well as removing any spuriously significant univariate interactions introduced by type II error.

## Results

### Investigating effects of nest-box type

The mean temperature and relative humidity found in the plastic nest boxes were 12.51°C (s.d. 2.73) and 79.64% (s.d. 21.54), and for the wooden nest boxes 12.59°C (s.d. 2.33) and 76.68% (s.d. 19.49), respectively. There was no significant difference in these variables between the plastic and wooden nest boxes (temperature: *P* = 0.917, relative humidity: *P* = 0.630).

In the 2014 breeding season 6 nests had a mass death of all chicks, these were all plastic nest boxes (2 ventilated, 4 non-ventilated) and they all contained blue tits. Yeasts were cultured from 36 of the chicks (*N* = 44) and 24 of the boxes (*N* = 25), including *Rhodotorula* species and various forms of white yeasts. Moulds were cultured from 15 of the 25 boxes swabbed and from 11 of the 44 chicks swabbed, and included *Aspergillus*, *Alternaria* and *Penicillium* species.

Fleas were present in 21 of the 25 nests and in very large numbers in some (maximum 2400). A total of only 6 ticks were found across 5 nest boxes of all types. Blowfly larvae were not present in any of the nests, but larvae of several non-parasitic species of fly were found, including high numbers of fannidae and muscidae larvae. Mites were present in 16 nests; 9 were plastic non-ventilated, 3 plastic ventilated and 4 were wooden boxes. Eurasian hornets were found in 5 of the nest boxes, all plastic, and egg laying in these boxes did not begin while the hornet was present. Of the 15 nest box swabs that cultured moulds, 10 were from blue tit nests (*N* = 15) and 5 from great tit nests (*N* = 10). Five swabs from blue tit chicks cultured moulds (*N* = 36) and 6 from great tit chicks (*N* = 31).

#### Univariate analyses

The number of flea larvae was significantly higher in great tit (mean = 452) than that in blue tit nests (mean = 200, *t*_18_ = 2.28, *P* = 0.035). Four chicks that had died between day 10 and fledging, and therefore identifiable by their rings, were recovered from nests. The dead chicks had a significantly higher score for total bacteria (*t*_8_ = −2.43, *P* = 0.04), enterobacterial (*t*_8_ = −3.12, *P* = 0.013) and fungal load (*t*_10_ = −2.33, *P* = 0.041) when previously swabbed, than the chicks that went on to fledge. These dead chicks had also weighed significantly less (*t*_45_ = 4.19, *P* < 0.001).

**Fig. 1.**
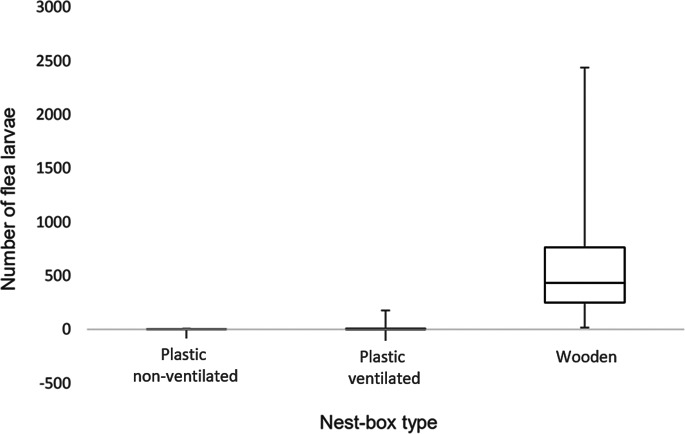
Numbers of flea larvae found in plastic non-ventilated, plastic ventilated and wooden nest boxes of blue tits *Cyanistes caeruleus* and great tits *Parus major*.

Numbers of flea larvae were significantly higher (*F*_2,21_ = 36.5, *P* < 0.001) in the wooden nest boxes compared to the plastic, see Fig. 1. Both plastic non-ventilated (ln mean 0.454, ±0.825, *P* < 0.001) and the plastic ventilated boxes (ln mean 1.41, ±2.04, *P* < 0.001) had dramatically fewer flea larvae than the wooden boxes (ln mean 591, ±1.38). Adult flea numbers were also significantly different between wooden and non-ventilated plastic boxes (*F*_2,21_ = 4.62, *P* = 0.022). Wooden boxes again contained significantly more (ln mean 3.01, ±1.68, *P* = 0.023) adult fleas than non-ventilated plastic boxes (ln mean 0.989, ±1.27).

To include all variables measured for multivariate analyses, only nests that went to completion were used. Nests that had PCR failure on their swabs were also removed from multivariate analyses, hence sample size varies between analyses.

#### Multivariate analyses

Nested analyses on the 4 chick level datasets showed a highly significant effect of box on chick enterobacterial load (*N* = 44, *F*_20,21_ = 11.4, *P* < 0.001), chick total bacteria (*N* = 44, *F*_20,21_ = 41, *P* < 0.001) and chick weight (*N* = 44, *F*_20,21_ = 7.97, *P* < 0.001), while box effect on chick fungal load was not quite significant (*N* = 44, *F*_20,21_ = 1.86, *P* = 0.083). Nested analyses including other variables broke down due to limitations of sample size and balance.

**Fig. 2.**
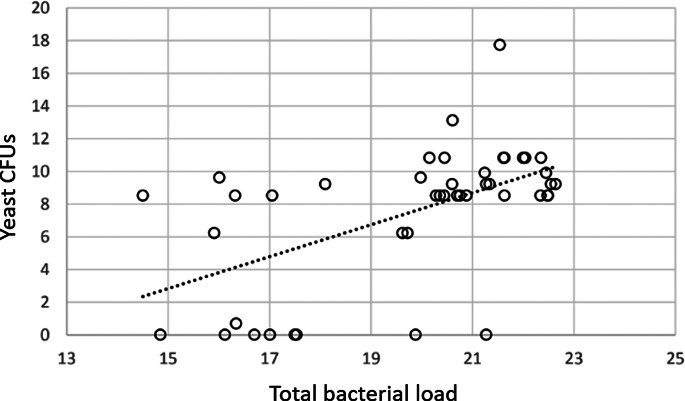
Total bacterial load by PCR (*x*) against yeast CFUs cultured (*y*) from blue tit *C. caeruleus* and great tit *P. major* chicks after swabbing (*R*^2^ linear = 0.31, *N* = 44, *P* = 0.002).

**Fig. 3.**
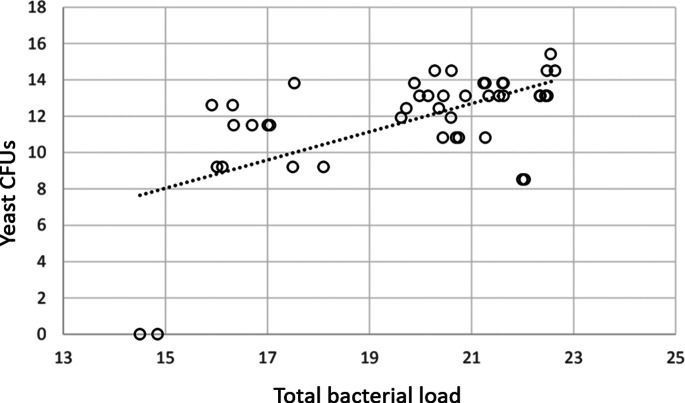
Total bacterial load by PCR on chicks (*x*) against yeast CFUs cultured from nest boxes (*y*) of blue tits *C. caeruleus* and great tits *P. major* after swabbing (*R*^2^ linear = 0.354, *N* = 44, *P* = 0.025).

Yeast load on the nest box was positively correlated with mean total bacterial load on chicks only (*N* = 24, *F*_1,20_ = 19.3, *P* < 0.001). Enterobacterial load on chicks was significantly positively correlated only with the total bacterial load on the box (*N* = 24, *F*_1,36_ = 6.01, *P* = 0.018). Total bacterial load on chicks was significantly positively correlated with enterobacterial load on the nest box (*N* = 24, *F*_1,37_ = 4.54, *P* = 0.04), and the yeast load: both on chicks (*N* = 44, *F*_1,37_ = 4.54, *P* = 0.041), Fig. 2 and the box (*N* = 24, *F*_1,37_ = 10.75, *P* = 0.002), Fig. 3. Bacterial load also varied significantly between types of nest box (*N* = 24, *F*_2,37_ = 4.07, *P* = 0.025), with wooden boxes having a higher total bacterial load (ln mean 20.5 ± 2.45) than plastic ventilated (ln mean 20.2 ± 2.16) and non-ventilated (ln mean 18.4 ± 2.08). The yeast load tended to be higher on chicks with higher total bacterial load, but this association was not significant (*N* = 44, *F*_1,37_ = 2.91, *P* = 0.097).

Wooden boxes (*N* = 20) had significantly higher occupancy rates, for the 2 species combined, compared to the plastic boxes (*N* = 46) (*χ*^2^ = 8.34, df = 2, *P* < 0.05) from 2011 to 2013. With regards to species differences in occupancy, over the 4 years monitored, 80% of the 40 great tit nests were in wooden boxes (*N* = 20), while 81% of the 117 blue tits nests were in plastic boxes (*N* = 46). Occupancy rates by year are shown in Table 1.

**Table 1.** Percentage of available plastic and wooden boxes occupied by blue and great tits in Betty Daw's wood 2011–2013

Year	2011	2012	2013	2014
Wooden boxes	70% (14)	75% (15)	80% (16)	65% (13)
Plastic non-ventilated boxes	61% (28)	74% (34)	52% (20)	29% (7)
Plastic ventilated boxes	n/a	n/a	n/a	64% (14)

Total number nested in that year in parentheses.

2011–2013 total available: plastic *N* = 46, wood *N* = 20. 2014 total available: plastic non-ventilated *N* = 24, plastic ventilated *N* = 22, wood *N* = 20.

Fledging success for blue tits was significantly higher in wooden than that in plastic boxes (*χ*^2^ = 152.7, df = 70, *P* < 0.05). No significant difference was found between plastic and wooden nest boxes for blue tit hatching success (*χ*^2^ = 16.7, df = 70, *P* > 0.05). There was no significant difference between hatching success for great tits between plastic and wooden boxes (*χ*^2^ = 2.48, df = 5, *P* > 0.05), nor in fledging success (*χ*^2^ = 2.75, df = 5, *P* > 0.05). Hatching and fledging percentage success rates are shown by year in Table 2 (blue tits) and in Table 3 (great tits).

**Table 2.** Percentage mean hatching (A) and fledging (B) success of blue tits *Cyanistes caeruleus* in plastic non-ventilated, wooden and plastic ventilated boxes in Betty Daw's wood 2011–2014

A			
	Plastic non-ventilated	Wood	Plastic ventilated
2011	95 (22)	96 (7)	n/a
2012	90 (20)	73 (4)	n/a
2013	95 (18)	97 (6)	n/a
2014	80 (12)	86 (5)	96 (10)

B			
	Plastic non-ventilated	Wood	Plastic ventilated
2011	93 (22)	86 (7)	n/a
2012	42 (20)	75 (4)	n/a
2013	78 (18)	96 (6)	n/a
2014	46 (12)	68 (5)	63 (10)

Total number of nests that year in parentheses.

**Table 3.** Percentage mean hatching (A) and fledging (B) success of great tits *Parus major* in plastic non-ventilated, wooden and plastic ventilated boxes in Betty Daw's wood 2011–2014

A			
	Plastic non-ventilated	Wood	Plastic ventilated
2011	100 (1)	83 (6)	n/a
2012	79 (2)	79 (7)	n/a
2013	100 (1)	70 (9)	n/a
2014	100 (2)	74 (9)	0 (0)

B			
	Plastic non-ventilated	Wood	Plastic ventilated
2011	100 (1)	94 (6)	n/a
2012	81 (2)	75 (7)	n/a
2013	50 (1)	58 (9)	n/a
2014	80 (2)	71 (8)	0 (0)

Total number of nests that year in brackets.

### Investigating the NPH

The *t*-tests showed none of the individual variables to be significantly different between herb and control nests. These included: total bacterial and enterobacterial load on both chicks and the nest box, brood size, nest mortality and flea and tick loads in the nest. Nested analyses showed a highly significant effect of box number on enterobacterial load (*F*_14,43_ = 4.5, *N* = 59, *P* < 0.001), but not that of total bacteria.

The load of enterobacteria on chicks was significantly and positively associated with the number of flea larvae in their nest (*F*_1,55_ = 9.8, *N* = 59, *P* = 0.003), their brood size (*F*_1,55_ = 16.9, *N* = 59, *P* < 0.001) and the presence of herbs (*F*_1,55_ = 15.8, *N* = 59, *P* < 0.001); with the herb group having a higher load of enterobacteria (6.35 ± 1.55) compared to the control group (5.69 ± 2.36). These analyses also included nest-level mortality rate and whichever bacterial type that was not the dependent variable, as they were correlated with each other in the earlier univariate correlations; however, neither was significant. Total bacterial load from swabs of the inside of the nest box was positively related to the number of hatchlings (*F*_1,11_ = 5.5, *N* = 14, *P* = 0.039). When nest mortality or any of the parasite counts were used as dependent variables, no factors were significantly associated.

## Discussion

Nest boxes have become highly important in avian conservation, not only for observing and manipulating behaviours of highly prevalent bird species, but also for the rearing and re-introduction of more threatened species, such as the highly threatened European storm petrels *Hydrobates pelagicus*, which gains protection against predatory sea gulls through artificial nest boxes (Libois *et al*., 2012). Better understanding of the benefits of different nest-box types would especially benefit these projects, as well as captive breeding programmes, where nest success is highly important.

Wooden boxes showed significantly higher occupancy rates than plastic nest boxes at our study site. When ventilation holes were introduced into a proportion of the plastic nest boxes, these showed higher occupancy rates the subsequent year than non-ventilated plastic boxes (Table 1). These ventilated plastic boxes also had higher fledging success rates than the non-ventilated plastic boxes (Table 2). Wooden boxes also had a significantly higher fledging rate for blue tits, fledging a higher percentage of chicks that hatched, than plastic boxes. This, however, is unlikely to be due to differences in nest-box microclimate as no significant difference was found in temperature and humidity between wooden and plastic nest-boxes. Despite this higher success rate, wooden boxes were also found to contain significantly higher numbers of fleas and a higher total bacterial load on chicks. For flea larvae this difference was substantial, with wooden boxes containing a mean number over 30-fold higher than the plastic boxes. There is the potential for wooden boxes to have built up a larger number of overwintering parasites as the wooden boxes were older than the plastic ones (Tomas *et al*., 2007). The boxes are, however, cleared out between each breeding season to prevent such accumulation, and the plastic boxes had been in use for 3 years prior to this study, so this difference is likely to be small. With the possibility of removal of nest material for analysis to occur the day after fledging, there is the potential for some parasites to migrate out of the nest box before this time. However with the flea larvae, which are the most starkly different, this is unlikely to be an issue with the pupae being non-mobile and the empty cocoons still being counted if they do emerge in this time. The large difference in parasite load between wooden and plastic nest boxes could pose major risks to chicks especially if nest boxes are erected in difficult to access sites, such as cliffs or mountains, for conservation purposes. Build-up of parasites year on year through overwintering is likely to become a major problem in such cases. Flea larvae are known to be pathogenic to chicks (Richner *et al*., 1993; Fitze *et al*., 2004) as are various species of bacteria (Davis *et al*., 1971); the chicks which died and had their rings recovered in this study had significantly higher bacterial loads than those that survived to fledging. Overall, however, the higher bacterial and parasite loads in wooden boxes did not appear to have affected fledging success, which – counterintuitively – was higher in wooden boxes.

Detrimental effects of higher parasite and bacterial load on chicks could have been compensated for by increased feeding by parents (Christe *et al*., 1996; Tripet and Richner, 1997). Assessment of parent health and weight across the breeding season, for plastic and wooden nest boxes, would provide an interesting insight into this – but quite difficult in a practical sense. It is also possible that the effects are not great enough to affect the measure of current fledging success used here, but instead be carried over to the following seasons. Detrimental effects may only appear when energy demands are greater; during the winter or future breeding attempts. These effects are also likely to be especially small in the man-made nest boxes used here, which are emptied between each season and so likely have much lower overwintering parasite and pathogen loads than natural nests. Overall lifetime reproductive success or survival across the following winter could be affected by the energy demands of the greater pathogen load in the nests birds were reared in. Future work could therefore assess longer term survival of chicks and future breeding success through re-trapping of returning individuals the following year. Sample size could become a problem for a study like this, with rate of individuals dying off or not returning the following year likely to be high. It could also be suggested that as wooden boxes have higher occupancy rates and there is likely competition for nest boxes between birds, that stronger individuals could out compete weaker individuals to nest in the wooden boxes. The greater fledging success in these boxes then could simply reflect the greater parental quality in them.

Nested models showed that box number had a highly significant effect on total bacterial load, enterobacterial load and the weight of chicks. Enterobacteriaceae are indicators of fecal contamination, and some are known pathogens of birds, so they were chosen to provide an interesting insight into contagion within nests and effects of brood size and other shared nest parameters on chicks. The correlation of bacterial load and chick weight within nest boxes most likely arises from shared nest-box characteristics such as species, nest-box type, brood size and cross-contamination between chicks. Nested analyses broke down when more factors were included; therefore it was not possible to test the effects of these covariates on nested models. The greater similarity of bacterial load on chicks within nests than between them does suggest the method used to swab chicks in the field was quantitative and well standardized. Lucas and Heeb (2005) carried out a brood manipulation experiment, moving chicks between nests for blue and great tits, and found that current box environment had a greater influence on chick bacterial levels than nest of origin or even species, illustrating the importance of nest-box environment and the parameters shared by all chicks in a nest on their bacterial load.

Other external factors must also be considered in determining the usefulness of plastic nest boxes, here in 2014 for instance Eurasian hornets were found in 5 of the nest boxes. These boxes were all plastic and in none of them did egg laying begin until after the hornets had left. Whether the presence of a hornet prevented the birds performing their normal behaviours remains to be investigated, but Lambrechts *et al*. (2008) have noted the presence of ants in nest boxes in Corsica affecting Paridae nesting. The likelihood of interactions like this becoming a major concern therefore needs to be considered.

If the NPH is valid, herbs could provide natural attenuation of higher parasite loads in wooden nest boxes. However, in the present investigation, herbs had no effect on parasite load, while enterobacterial load was actually greater in herb nests. In any case it seems unlikely, due to differences in climate and therefore availability of these plants that this behaviour will evolve in temperate areas in the near future. In the present study, aromatic plants previously demonstrated to have effects on nest parasitism, which do not grow naturally in the UK, were bought commercially, which could have the potential for contamination with extraneous bacteria, pesticides or herbicides. It is also possible that environmental temperatures may have been insufficient to volatilize plant compounds in the nest as the behaviour in this species has only been reported in much warmer Mediterranean regions of Europe; mainly Corsica (Banbura *et al*., 1995; Lambrechts and Dos Santos, 2000; Petit *et al*., 2002; Mennerat, 2008) and Portugal (Pires *et al*., 2012). The average temperature inside nest boxes in a study by Garcia-Navas *et al*. (2008) in central Spain was 16–18°C, while in the present study site in 2014 it was around 12°C. A review by this author of studies experimentally testing the NPH showed that most studies were compromised by low sample size and confounding variables (Scott-Baumann and Morgan, 2015). In the present study, as many variables were accounted for as possible, but as more variables are included, so sample size must also increase to maintain power. With the data from the nest-box material comparison, which was conducted the following year, we now know how much lower the parasite and pathogen load can be in the plastic boxes. With the wooden boxes having a greater bacterial and parasite load these could provide a better platform to assess the NPH, than the plastic ones used here.

Differences clearly existed in the occupation rate of different nest boxes: blue tits nested mostly in plastic boxes and great tits mostly in wooden. Despite this blue tit preference for plastic nest boxes, their fledging success was significantly higher in wooden boxes. However with the great tit being a larger species and on average nesting earlier in the UK than blue tits (BTO, 2021), it is possible that this apparent preference by blue tits was simply due to being out-competed for the wooden boxes. This could therefore leave the plastic nest boxes for the blue tits and explain the differences in breeding success. Although no difference in nest-box temperature or humidity was found between plastic and wooden boxes, a more comprehensive assessment of the dynamic ranges of these conditions in the nest boxes could be made. Relative humidity especially is highly variable and difficult to reliable record therefore continuous measurements could be made through the use of a data logger probe, and thus provide a larger number of data points and a more reliable mean. This kind of assessment would also have the added benefit of assessing temperature and humidity while the boxes are actually being actively nested in, given the nest material and fledglings themselves could have a potential impact on those parameters. The stark differences in parasite load between plastic and wooden boxes still remains to be explained. It is possible that if temperature and humidity change significantly during the nesting period, this could then perhaps mean the environment inside the nest boxes actually becomes inappropriate for parasite development. If so then those parameters could also pose inappropriate conditions for the chicks to develop in. These possible inappropriate conditions in the plastic boxes could then explain both the lower fledging success rate and the simultaneous lower parasite and bacterial load. The increase in occupancy rates and the increased fledging success rate after the addition of ventilation holes in the plastic nest boxes is also interesting, and could provide a very simple remediation process that could be performed to improve the design of plastic nest boxes. This is an important assessment to make as some conservation charities currently recommend against the use of plastic nest boxes based on concerns regarding issues with condensation, but grounded in no published scientific evidence that we can find.

This report is one of few investigations of its kind into assessing the conditions inside plastic nest boxes as compared to the traditional wooden boxes. Further work is required to more comprehensively assess the suitability of different nest-box materials before their large-scale use. Some parameters of nest success are higher for wooden boxes, while lower parasite and bacterial loads can be seen in plastic boxes. Greater scientific attention to the characteristics of nest box design and material and their impact on disease burden and nest success is needed in general before recommendations are made on their use. In this case in particular a more dynamic assessment of nest-box microclimate throughout nesting season would be pertinent before final recommendations are made.
